# A Review of Nutrients and Compounds, Which Promote or Inhibit Intestinal Iron Absorption: Making a Platform for Dietary Measures That Can Reduce Iron Uptake in Patients with Genetic Haemochromatosis

**DOI:** 10.1155/2020/7373498

**Published:** 2020-09-14

**Authors:** Nils Thorm Milman

**Affiliations:** Department of Clinical Biochemistry, Næstved Hospital, University College Zealand, DK-4700 Næstved, Denmark

## Abstract

**Objective:**

To provide an overview of nutrients and compounds, which influence human intestinal iron absorption, thereby making a platform for elaboration of dietary recommendations that can reduce iron uptake in patients with genetic haemochromatosis.

**Design:**

Review. *Setting*. A literature search in PubMed and Google Scholar of papers dealing with iron absorption.

**Results:**

The most important promoters of iron absorption in foods are ascorbic acid, lactic acid (produced by fermentation), meat factors in animal meat, the presence of heme iron, and alcohol which stimulate iron uptake by inhibition of hepcidin expression. The most important inhibitors of iron uptake are phytic acid/phytates, polyphenols/tannins, proteins from soya beans, milk, eggs, and calcium. Oxalic acid/oxalate does not seem to influence iron uptake. Turmeric/curcumin may stimulate iron uptake through a decrease in hepcidin expression and inhibit uptake by complex formation with iron, but the net effect has not been clarified.

**Conclusions:**

In haemochromatosis, iron absorption is enhanced due to a decreased expression of hepcidin. Dietary modifications that lower iron intake and decrease iron bioavailability may provide additional measures to reduce iron uptake from the foods. This could stimulate the patients' active cooperation in the treatment of their disorder and reduce the number of phlebotomies.

## 1. Introduction

Genetic haemochromatosis is characterized by an increased intestinal dietary iron uptake, of both nonheme and heme iron [1–3], which in the long term may lead to the gradual accumulation of excess iron in the body and clinical symptoms of iron overload. The various forms of genetic haemochromatosis are caused by mutations on different iron regulatory genes and are divided into two main groups: *HFE*-haemochromatosis being caused by mutations on the *HFE*-gene on chromosome 6 and non-*HFE*-haemochromatosis. Among people of northwestern European descent, including ethnic Danes, *HFE-*haemochromatosis is the most common variant [4, 5], while non-*HFE*-haemochromatosis occurs sporadically [6].

Homozygosity for a particular gene means that the two alleles of the gene present on both homologous chromosomes are identical. Homozygosity for the *HFE*-C282Y mutation (C282Y/C282Y) is the leading cause of preclinical and clinical haemochromatosis in ethnic Danes, where more than 95% of the patients have this mutation [7]. Heterozygosity for a particular gene means that the two alleles of the gene present on both homologous chromosomes are not identical, i.e., the cells contain two different alleles, one mutant and one wild-type allele. Heterozygosity for the C282Y mutation (C282Y/wild type) is generally associated with a normal iron absorption pattern for both nonheme and heme iron [8]. Population studies have shown that among ethnic Danes, at least 0.4%, or one in 250, are C282Y homozygous equivalent to more than 20,000 people in Denmark, and more than 500,000 people are heterozygous (C282Y/wild type) or compound heterozygous (C282Y/H63D) for the *HFE*-mutations [9]. In comparison, the UK Biobank study examined more than 450,000 individuals of European descent and found a prevalence of C282Y homozygosity of 0.6%, or one in 167 persons [10].

Hepcidin, which is produced in the liver, is considered to be the “master regulator” of body iron homeostasis, and one of its main tasks is to inactivate ferroportin, which regulates iron transport out of the cells (efflux) through the cell membrane in enterocytes, hepatocytes, and macrophages [11]. Normal *HFE-* and transferrin receptor-2 complexes on the cell membrane of the hepatocytes stimulate the production/activation of hepcidin, which subsequently inhibits intestinal iron uptake [11, 12]. In haemochromatosis, because of a defective *HFE*-complex, the production/activation of hepcidin is reduced, resulting in an increased intestinal iron uptake, which by and large is independent of the body's iron status. *HFE*-haemochromatosis and several other forms of genetic haemochromatosis are characterized by a low plasma concentration of hepcidin and a condition termed “hepcidin insufficiency” [11, 12].

The standard treatment of *HFE*-haemochromatosis consists of repeated lettings of 400–500 ml of whole blood (phlebotomy) where excess body iron is gradually removed [4, 13]. Treatment is divided into the induction phase, where patients are phlebotomized weekly or every other week until serum ferritin has declined to 50–100 *µ*g/L. In the following maintenance phase, phlebotomy is performed two to four times a year maintaining ferritin around 50–100 *µ*g/L [4].

In haemochromatosis, body iron overload is gradually increasing over time due to excessive absorption of iron from the food. Consuming a diet void of iron or with a low iron content will downregulate the body iron accumulation. Another important factor is the bioavailability of the iron in the food as well as the presence of promoters *versus* inhibitors of iron absorption. There exists only scarce literature concerning the significance of dietary iron intake in haemochromatosis, and the effect of dietary intervention has not been sufficiently investigated [3] However, the focus on population groups with specific dietary habits has provided us with valuable data showing that the content and the chemical form of iron in the diet have a definite influence on body iron status. Vegetarians/vegans have a low body iron status and many, especially women in the reproductive age, develop iron deficiency [14, 15]. In a cross-over study, total and fractional iron absorption was significantly lower in a vegetarian than in a meat-rich diet [16]. A Western-type vegetarian diet has a low bioavailability ranging from 5 to 12% [17].

The purpose of this paper is to review the existing knowledge on the different nutrients and compounds that influence iron uptake from the diet.

## 2. Methods

This paper is based on literature searches in the PubMed and Google Scholar databases as well as literature references cited in published articles, review papers, and books on iron metabolism. Search terms included “iron absorption” and “iron absorption [AND] … name of the specific nutrient or compound”. Primarily, human studies on iron absorption have been included, but where necessary, some *in vitro* and animal studies have been included for the purpose of clarification.

### 2.1. Dietary Iron Intake

The composition of the diet concerning the content of iron and the content of inhibitors/promoters of iron absorption may have a significant influence on the development of clinically overt haemochromatosis.

The recommended intake of dietary iron in healthy men in the Western World varies from 8 to 11 mg/day, depending on country-specific recommendations [18–20]. Danish men have a median dietary iron intake of 12.7 mg/day (10–90 percentile 8.3–18.0) [21], indicating that the majority have an intake which is above the recommended intake. In the USA, the “estimated” mean iron intake is even higher, 16.1 mg/day [22].

In addition, men have a distinctly higher intake of meat [21, 23] and alcohol [21] than women, both factors, which increase iron uptake (see below) and the rate of body iron accumulation in men with genetic haemochromatosis.

In healthy women of reproductive age, the recommended iron intake varies from 15 to18 mg/day [18–20]. A review of dietary iron intake in women in Europe has recently been published [24]. Danish women in the reproductive age have a median iron intake of 9.7 mg/day (5–95 percentile 5.6–14.5) [21, 24], indicating that all women have an intake below the recommended intake. In the USA, the “estimated” mean iron intake is higher, 12.3 mg/day [22].

The majority of healthy women of reproductive age have an iron intake which is markedly below the recommended intake [24]. In addition, women of reproductive age have physiological iron losses by menstruations [25] and pregnancies [26], exerting an inhibitory effect on body iron accumulation in women with haemochromatosis, thereby delaying or preventing the onset of clinical disease.

Although the HFE-mutations occur with the same frequencies in men and women, preclinical and clinical haemochromatosis is much more prevalent in men than in women and presents at a younger age in men compared to women [4]. This gender difference is partly explained by a higher dietary iron intake in men, combined with a higher intake of absorption promoters, e.g., meat and alcohol, compared to women.

### 2.2. Different Forms of Dietary Iron

**Figure 1:**
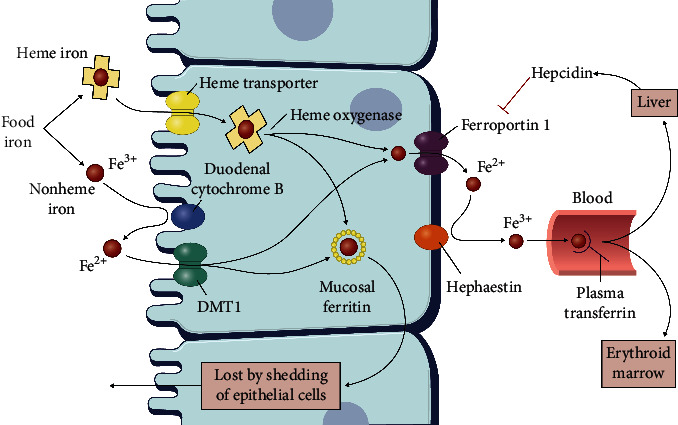
Mechanisms of intestinal iron absorption. Most iron is absorbed in the duodenum and proximal jejunum due to the acidic pH of the intestinal content. More, distally in the jejunum, pH becomes neutral or alkaline, and iron uptake declines. Nonheme food iron Fe^3+^ enters the nonheme iron pool and heme iron enters the heme iron pool. Fe^3+^ is reduced to Fe^2+^ by duodenal cytochrome B and subsequently enters the luminal side of the enterocyte by the iron importer DMT-1 pathway. Fe^2+^ is transferred to the basolateral side of the enterocyte, effluxed by the iron exporter ferroportin, and subsequently oxidized to Fe^3+^ by hephaestin and transferred to the carrier protein transferrin in the blood plasma. Part of the iron enters the intracellular ferritin iron pool and is lost by desquamation of the enterocyte into faeces. Hepcidin from the liver inactivates ferroportin, thereby inhibiting iron uptake. Heme iron is absorbed within the intact protoporphyrin ring by a separate pathway possibly involving a heme transporter and heme oxygenase. Figure adapted with permission from [27].

An overview of the intestinal absorption of nonheme and heme iron is shown in Figure 1.

Iron in the foods exists in two forms: (i) inorganic iron = nonheme iron consisting of mostly ferric iron (Fe^3+^) and some ferrous iron (Fe^2+^) and (ii) organic iron consisting of heme iron from animals and ferritin iron from animals and plants. Most iron in the foods consists of inorganic ferric iron, which is chemically relatively inactive. Ferric iron must be reduced to ferrous iron either by gastric acid pH, reducing components in the meal, or by the ferric reductase enzyme duodenal cytochrome b, which catalyzes the reduction of Fe³⁺ to Fe^2^⁺, before the ferrous iron can be taken up in the enterocytes by the protein dimetal transporter-1 (DMT-1) pathway [28]. Ferric iron may be taken up by the mucin-beta3-integrin-mobilferrin pathway (IMP) [29], but the relative physiological and nutritional importance of the IMP compared with the DMT-1 pathway in the absorption of iron in humans is not known.

Heme iron, which is ferrous iron present in haemoglobin in the red blood cells and myoglobin in the myocytes, is absorbed within the intact protoporphyrin molecule threefold to fourfold more efficiently than inorganic ferric iron. Heme iron absorption is relatively independent of the effects of various promoters/inhibitors of iron absorption in the food matrix (except for calcium, see below) [30].

The ferric iron contained in the ferritin molecule, i.e., “ferritin iron” from animal and plant foods, is released from the ferritin shell by the gastric acid pH as well as by cooking. It enters the common nonheme iron pool in the meal and is absorbed as other nonheme iron compounds [31, 32].

### 2.3. Promoters and Inhibitors of Iron Absorption

Table 1 gives an overview of the principal known promoters and inhibitors of food iron absorption. When giving dietary recommendations to patients with haemochromatosis, it is essential to emphasize that promoters and inhibitors of iron uptake must be ingested in the same meal as the food iron, in order to exert their effects.

## 3. Promoters

### 3.1. Acids

#### 3.1.1. Gastric Acid

The gastric secretions contain hydrochloric acid with a low pH, which promotes the reduction of ferric iron in the foods to ferrous iron. Gastric acid is one of the most important luminal factors being mandatory for an optimal nonheme iron absorption. In persons with histamine-fast achlorhydria, adding hydrochloric acid to a solution of ferric chloride increased iron absorption more than fourfold, but had no effect on heme iron absorption [33]. Conversely, inhibition of gastric acid production by histamine H_2_-receptor blockers caused a marked decrease in iron absorption [34]. Administration of an antacid in association with a meal likewise reduced iron absorption significantly [34] and patients with achlorhydria often develop iron deficiency [35].

#### 3.1.2. Organic Acids

Ascorbic acid = vitamin C is a potent reducing and antioxidant agent, found in fruits and vegetables [36]. Among the organic acids, ascorbic acid possesses the most pronounced enhancing effect on nonheme iron absorption [37–39]. Ascorbic acid forms a soluble chelate with ferric iron, which prevents the formation of insoluble and unabsorbable iron compounds and it is assumed that ascorbic acid facilitates the reduction of ferric to ferrous iron [40]. In contrast, an *in vitro* study on the human epithelial Caco-2 cell line showed that ascorbate increased apical ferric iron uptake in a concentration-dependent manner with a significant difference between iron uptake and iron reduction; ascorbate enhanced the uptake of ferric chloride iron through the formation of a Fe^3+^-ascorbate complex [41]. Therefore, the mechanisms of the enhancing effect of ascorbic acid on iron uptake are still not fully clarified.

Addition of 15 mg ascorbic acid, which is commonly found in vegetables, improved the geometric mean nonheme iron absorption from a basic rice meal with 98% [42]. The effects of the chemical composition of fruit juices and fruits on the absorption of iron from a basic rice meal were examined in 234 women, using the erythrocyte utilization of the double isotope radioactive iron method. The corrected geometric mean iron absorptions with different fruit juices were closely correlated with the ascorbic acid contents of the juices [43], and there was a close correlation between iron absorption from various fruits and their ascorbic acid content [42].

Adding ascorbic acid to a test meal with a molar ratio of ascorbic acid : iron of 1.6 : 1 significantly increased iron absorption [43], and adding ascorbic acid to a test meal with a molar ratio ascorbic acid : iron of 4 : 1 increased nonheme iron uptake with 185% [44].

The facilitating effect of ascorbic acid on iron absorption from a complete diet is less pronounced than that from single-test-meal studies [45]. This may explain why several studies did not show a significant effect on iron status after prolonged supplementation with ascorbic acid [46]. However, in the complete diet study, there was still a significant correlation between the intake of ascorbic acid and iron uptake [45]. Probably, if the daily diet contains adequate amounts of heme iron and meat factors from animal meat, which are strong promoters of iron uptake, the enhancing effect of ascorbic acid may hardly be discernable.

The European Food Safety Authority has reviewed the association between ascorbic acid and iron uptake in 2010 and 2014 and concluded that “vitamin C contributes to increasing nonheme iron absorption” [47, 48].

#### 3.1.3. Citric Acid, Malic Acid, and Tartaric Acid

Citric acid, malic acid, and tartaric acid are present in many berries, fruits, and vegetables. Citric acid seems to have a promoting effect on nonheme iron absorption, possibly through the reduction of ferric to ferrous iron. Although citric acid is found in fruits of which the majority also contain ascorbic acid, studies indicate that citric acid *per se* stimulates iron absorption [42, 43]. However, one human single-test-meal study showed that adding citric acid to the meal decreased iron uptake [38].

The results concerning malic acid are also controversial. One study showed that adding malic acid to a rice meal significantly increased iron uptake [42] while a subsequent study from the same group found an inhibitory effect of fruits with high malic acid content [39].

Tartaric acid from citrus fruits and wine grapes appears to have a moderate stimulatory effect on nonheme iron absorption [42].

#### 3.1.4. Lactic Acid

Lactic acid is produced by fermentation of carbohydrates by cultures of lactic acid bacteria (mainly Lactobacillus species and some Streptococcus species). Lactic acid fermentation of specific food items increases the bioavailability of iron [49].

In lactic acid fermented milk products, lactic acid is produced from lactose and the fermentation process has as a lowering effect on pH in all kinds of foods [50]. Galan et al. [51] measured iron absorption from a typical French meal and from the same meal after the addition of a glass of skimmed milk or plain yogurt. Iron absorption was similar in the three meals, about 2.1% [51].

In women consuming a typical plant-based, phytate-rich Mexican meal once daily, the addition of 250 ml skimmed milk or 150 g yogurt to the meal for 13 days did not change iron uptake compared to the same meal without milk products; uptake was also similar in the diet with milk and the diet with yogurt [52]. Under real-life conditions, a moderate intake of dairy products probably has no significant effect on iron absorption [51, 52].

The lactic acid fermented cabbage sauerkraut has a high lactic acid content and an enhancing effect on iron absorption compared to nonfermented vegetables [42].

Lactic acid is an effective promoter of nonheme iron absorption, both by itself and in lactic acid-containing gruels made from maize and sorghum mask for beer production [53]; lactic acid enhanced iron absorption both in gruels and in the final brews [53].

In an *in vitro* study using Caco-2 cells, lactic acid fermentation of maize products as well as the addition of small amounts of lactic acid to unfermented maize products improved iron bioavailability [54]. Nongenetically modified maize contains phytate but has a low intrinsic content of phytase [55], so the enhancing effect of lactic acid is hardly due to the activation of intrinsic phytase.

Another study combining *in vitro* Caco-2 cells and an *in vivo* mouse model showed that lactic acid fermentation of bread enhanced iron absorption both *in vitro* and *in vivo* [56].

Lactic acid fermentation of cereal flours [57] and whole-grain wheat bread [58] reduces the phytate content between 47 and 100% [57], depending on the phytase content of the specific grain [57]. A lactic acid fermented, phytate-rich, oat gruel increased nonheme iron absorption significantly, compared with a nonfermented gruel [59].

Lactic acid sourdough fermentation was more efficient than yeast fermentation in reducing the phytate content in whole-grain wheat bread, 62% versus 38% reduction, respectively. Furthermore, lactic acid bacteria present in sourdough induce acidification, leading to increased magnesium and probably also iron solubility [58].

Lactic acid fermentation can reduce the phytate content in foods in two ways: some species of lactic acid bacteria by themselves produce phytase [60], and in addition, the acid fermentation process can activate the intrinsic but inactive phytase in the cereals [57].

Altogether, there is good evidence that lactic acid has an enhancing effect on nonheme iron absorption and that lactic acid fermentation of selected food items increases iron uptake both directly and indirectly in cereal products by reducing the content of phytate.

#### 3.1.5. Meat Factors

Meat factors are present in meat from mammals, birds, and fish. It has been recognized for many years that the fractional iron absorption, i.e., percent iron absorbed of the total iron content, is significantly higher from animal foods than from vegetable foods [38, 61].

Cook and Monsen [62] studied the effects of different proteins of animal origin on iron uptake in humans. In a semisynthetic meal, substituting beef, lamb, pork, chicken, liver, and fish for egg ovalbumin resulted in a twofold to fourfold increase in iron absorption [62]. Pork meat increased iron absorption significantly from a 5-day controlled diet, when compared to a vegetarian diet with similar vitamin C and phytic acid content, clearly demonstrating the enhancing effect of the meat factors [16].

Hurrell et al. [63] tried to characterize the meat factors. Freeze-dried beef and chicken muscle increased nonheme iron absorption by 180% and 100%, respectively, relative to ovalbumin. The enhancing effect of muscle tissue on iron absorption was protein and/or peptide related, but other factors might play a role [63].

#### 3.1.6. Heme Iron

Heme iron is present in haemoglobin in the red blood cells and in myoglobin in the muscle cells. The metallo-protoporphyrin heme contains one ferrous iron atom. Pure heme iron, i.e., heme without its protein (globin) moiety, is poorly absorbed, probably because it forms macromolecular polymers [64]. The absorption mechanisms of heme are not fully clarified; it appears that heme is taken up by the enterocyte as an intact molecule and subsequently degraded by the enzyme heme oxygenase, thereby releasing the iron [30].

Compared to nonheme iron, the ferrous iron incorporated in the heme-globin molecule is much more easily absorbed, and heme in association with its globin moiety can therefore be considered a strong “facilitator” of iron uptake. Even if heme iron constitutes only a smaller part of the total iron content in the meal, the fractional absorption is fourfold to sixfold higher [65–68]. Dietary iron is predominantly nonheme iron with about 10–15% in the form of heme iron in diets containing animal meat. Heme is highly bioavailable, about 20–30% of heme iron being absorbed [28]. Absorption of nonheme iron is more variable and significantly affected by promoters/enhancers of iron uptake in the diet with 1–10% of nonheme iron being absorbed [28]. Heme iron is therefore the “super iron” for healthy subjects and the “poisonous iron” for persons with haemochromatosis.

#### 3.1.7. Alcohol

The influence of alcohol (ethanol) on body iron status seems to be complex. It has for a long time been recognized that regular and/or excessive consumption of alcohol predisposes to iron overload [69]. In population studies of normal subjects, there is a significant association between alcohol intake and the iron status marker serum ferritin, in both men and women. An increased alcohol intake is associated with an increase in ferritin and body iron content [70–72]. In patients with *HFE*-haemochromatosis, excessive alcohol consumption accentuates the biochemical and clinical disease expression and therefore the risk of liver cirrhosis and liver cancer [73]. Toxicity of alcohol in combination with iron accumulation in the liver may have deleterious effects on inflammation, fibrogenesis, and carcinogenesis [73].

In one single-dose study, there was no effect of alcohol on the absorption of ferrous ascorbate and haemoglobin iron [69], but a significant enhancement of absorption of ferric chloride. This effect could be due to the alcohol-induced stimulation of gastric acid production [69]. Another single-dose study showed that the acute ingestion of ethanol did not influence the absorption of inorganic iron, while it diminished the absorption of heme iron [74].

However, the enhancing influence of alcohol on iron absorption may be indirect, relying on an inhibition of hepcidin synthesis in the liver. In patients with alcoholic liver disease, serum prohepcidin concentrations were significantly lower than in healthy subjects, and concentrations were most decreased in patients with the highest serum ferritin values. In an ethanol-fed mouse model, hepatic hepcidin-1 mRNA expression was significantly lower than that in controls. Prohepcidin was expressed in the cytoplasm of hepatocytes of mice liver tissue sections, and the expression decreased after ethanol loading [75].

Other animal studies have confirmed these findings [76–78]. It appears that alcohol loading downregulates hepatic hepcidin expression and thereby leads to an increase in intestinal iron absorption. Both acute and chronic alcohol exposures suppress hepcidin expression in the liver [79].

Maybe the “missing link” between alcohol consumption and increased iron absorption is predominantly the alcohol-induced inhibition of hepcidin expression and not as earlier presumed being due to effects on the absorptive processes in the intestine. However, patients with haemochromatosis already have a low hepcidin level and it has not yet been clarified whether alcohol will contribute to a further reduction of hepcidin.

## 4. Inhibitors

### 4.1. Gastric Acid Inhibitors

There exist two types of gastric acid inhibitors, histamine H_2_-receptor antagonists, e.g., cimetidine, and proton pump inhibitors, e.g., lansoprazole. Both types of medication can effectively reduce the production of gastric acid [80, 81]. In a nested case-control study, among subjects without known risk factors for iron deficiency, the use of a gastric acid inhibitor for two years or more was associated with an increased risk of iron deficiency. The risk increased with increasing potency of acid inhibition and decreased after medication discontinuation [82].

In patients with *HFE*-haemochromatosis, consecutive measurements of serum iron demonstrated a 50% decrease in the area under the curve (AUC) from a test meal after 7 days of proton pump inhibitor therapy, indicating decreased absorption of nonheme iron [83]. Furthermore, treatment of patients with genetic haemochromatosis with proton pump inhibitors caused a significant reduction in the volume of blood that had to be drawn annually, in order to maintain serum ferritin in therapeutic levels [83].

A similar finding was observed in a retrospective analysis of patients with *HFE*-haemochromatosis, where treatment with proton pump inhibitors for two years or more significantly reduced the number of phlebotomies required to maintain serum ferritin levels below 100 *μ*g/L [84]. These studies show that gastric acid inhibitors decrease iron absorption in both iron-depleted and iron-overloaded individuals.

### 4.2. Antacids

Antacids are used for immediate relief of gastroesophageal reflux of gastric acid giving unpleasant symptoms called pyrosis, cardialgia, or heartburn. Antacids are normally used on a demand basis, seldom for long-term treatment. Many preparations contain calcium carbonate and magnesium oxide, magnesium hydroxide, or magnesium trisilicate. If taken in close association with a meal, this kind of medicine can inhibit iron uptake from the food in three ways: (i) by neutralizing the gastric acid pH [33], (ii) by calcium-induced inhibition of iron absorption (see Section 4.9 and [85]), and (iii) by a possible inhibition of magnesium compounds on iron absorption [86, 87].

### 4.3. Oxalic Acid-Oxalates

Oxalic acid and oxalates are common in plant foods, and the largest amounts are found in rhubarb, spinach, mangold, and purslane [88]. Oxalic acid may be present as insoluble calcium or magnesium oxalate crystals or as soluble sodium or potassium oxalate. In humans, approximately 75% of all urinary tract stones are composed of calcium oxalate, and hyperoxaluria is a primary risk factor for urolithiasis [89].

Oxalate is a well-known inhibitor of calcium absorption in humans due to the poor solubility of calcium oxalate [90]. It is a widespread perception among nutritionists/dieticians that oxalic acid is a strong inhibitor of iron absorption. This is stated (without evidence) in dietary publications and cookbooks (e.g., [91, 92]). It may rely on the fact that ferric oxalate is slightly soluble and ferrous oxalate is poorly soluble in water.

Calcium oxalate appears to depress iron absorption in some circumstances—the addition of 1 g calcium oxalate to a cabbage meal was associated with a 39% depression in iron absorption [42]; this is surprising as calcium oxalate is almost insoluble and therefore should be chemically inert. However, there was no relationship between the oxalate content and iron absorption when vegetables that contained large amounts of oxalate were examined; iron absorption was poor from spinach and beetroot greens but good from beetroot [42]; these findings could be explained by the high polyphenol and calcium content in spinach, the high polyphenol content in beetroot greens, and the low polyphenol and high citric acid and ascorbic acid content in beetroot [42].

The influence of oxalic acid/oxalate on iron absorption may depend on the chemical state of the oxalate in the food. Spinach is rich in oxalic acid/oxalate but also rich in calcium; if the oxalate exists in the form of calcium oxalate, then it should not be able to bind ferric iron in the food and therefore have a minor influence on iron absorption.

In a test meal consisting of wheat bread rolls and kale with a low content of oxalic acid, the addition of soluble potassium oxalate to the meal did not have any negative influence on iron absorption [93]. Absorption from the test meal with bread rolls and spinach was lower than that from the meal with bread rolls and kale (without added potassium oxalate), possibly due to the higher content of polyphenols and calcium in the spinach meal compared to the kale meal [93]. Consequently, oxalic acid in plant foods does not inhibit iron absorption and does not contribute to the reported inhibitory effect of spinach on iron uptake [93].

### 4.4. Phytic Acid-Phytates

Phytic acid, the hexaphosphate of myo-inositol, is a bioactive compound widely distributed in plant foods [94]. Phytic acid has an affinity to form complexes with polyvalent cations including iron, in this way interfering with intestinal absorption. The main sources of phytate in the daily diet are cereals and legumes as well as oilseeds and nuts [94]. These foods are important in the human diet and represent approximately 40% of the caloric intake for humans in developed countries [95].

Phytates are strong inhibitors of iron absorption in a dose-dependent manner [42, 96–100]. Furthermore, the inhibitory effect of phytates on iron absorption can be reversed by ascorbic acid and to a lesser degree by meat factors [97, 98], indicating that ascorbic acid has a higher affinity to ferric iron than phytate.

The clinical impact of the phytate content in bran on iron absorption is evident. In vegetarians/vegans [14–17] and in young women, long-term daily intake of fiber-rich wheat bread with a high fraction of whole grain decreased body iron status (serum ferritin) [100].

Lactic acid fermentation causes a marked reduction in the phytate content of cereal flours [57] and lactic acid sourdough fermentation is more efficient than yeast fermentation in reducing the phytate content in whole-grain wheat bread [58]. Reduction of the phytate content has an enhancing effect on iron absorption [42, 96–101].

Lactic acid fermented oat gruel increased nonheme iron absorption significantly compared with a nonfermented gruel, due to the reduction in phytate content [59]. Further details of the interactions between lactic acid fermentation, phytate, and phytase are given in the above section on lactic acid.

### 4.5. Polyphenols

Polyphenols are widely present in the human diet as components of fruits, berries, vegetables, spices, pulses, and whole grains, and they are especially high in tea, coffee, cocoa, red wine, and some herb teas [102, 103].

Phenolic compounds (phenolic monomers, polyphenols, e.g., tannic acid and tannins) inhibit iron uptake by a complex formation of chelates with iron in the gastrointestinal lumen [104, 105], making the iron less available for absorption [42, 98, 99, 106–109]. The inhibitory effect of polyphenols on iron uptake [42, 99, 100, 104–109] is dose-dependent [107–109].

Population studies of healthy Danish subjects (nonblood donors) showed significant negative correlations between body iron status (serum ferritin) and coffee *plus* tea consumption in men [71] but not in women [71].

A cup of tea reduced iron absorption from a test meal by 64% and a cup of coffee by 39% [107]. In patients with haemochromatosis on maintenance phlebotomy treatment, the content of polyphenols in one cup of black tea consumed with each meal has been shown to inhibit iron uptake and prolong the phlebotomy intervals [110]. Another study reported that beverages containing 20–50 mg total polyphenols per serving reduced iron absorption from a bread meal by 50–70%, whereas beverages containing 100–400 mg total polyphenols per serving reduced absorption by 60–90%. The inhibition by black tea ranged from 79 to 94% [109]. Probably due to its content of polyphenols, the widely used spice, chili, significantly inhibits iron absorption from a test meal [111].

However, the inhibitory effect of polyphenols on iron absorption can be counteracted and abolished by adequate doses of ascorbic acid added to the meal [98], suggesting that ascorbic acid has a higher affinity to ferric iron than polyphenols.

Tannins are polyphenolic compounds that are found in many fruits, berries, and legumes. Principal dietary sources of tannins are pomegranate juice, tea, coffee, dark chocolate, red wine grapes, and red wine, especially red wine, which has been aged in oak barrels [102].

Tannic acid and tannins are potent inhibitors of iron absorption. Adding tannic acid to a vegetable meal inhibited iron absorption in a dose-dependent manner [42]; 5 mg tannic acid in a meal inhibited absorption by 20% and 100 mg by 88% [108].

### 4.6. Soya Bean Protein

Soya beans are the largest global protein crop and the most efficient source of plant protein per hectare cultured area. Soya beans are used to make a variety of vegetarian foods such as soya milk, tofu, and edamame, as well as miso, tempeh, and natto from fermented soya beans. Most of the soya bean crops are processed into soya bean flour and soya bean oil, and soya bean products are used worldwide as a protein-rich nutrient [110]. The iron content in soya beans shows considerable variation. Some beans can be rich in iron and contain as much as 15.7 mg/100 g [113], while other beans have a lower iron content varying from 5.1 to 4.0 mg/100 g [114, 115].

Most of the iron is in the form of organic plant ferritin iron, which by itself has a reasonably good bioavailability [31, 32]. However, nongenetically modified soya beans are also rich in phytic acid, which inhibit iron absorption [99], and in addition, they have a low intrinsic content of phytase [55]. Removing the phytates from soya bean flour increased iron absorption markedly, but absorption was still significantly lower than from the egg white control meal, suggesting that other intrinsic factors in the flour inhibit iron uptake [99]. Moreover, another study showed that different kinds of processed soya bean proteins *per se* appear to impair iron uptake [116]. In humans, the unmodified soya bean protein isolate markedly inhibited iron absorption and there are two major inhibitors of iron absorption in soya bean protein isolates, phytic acid and a protein-related moiety contained in the conglycinin fraction [117]. In contrast, another study [38] found that processed soya bean flour added to a test meal increased iron absorption significantly—which the authors presumed could be explained by the high iron content in the flour [38].

Soya beans are rich in calcium, the content varying from 150 to 277 mg/100 g [113, 115] compared to 126 mg/100 g in partly skimmed milk [113, 115]. The calcium content can also contribute to a decrease in iron absorption (see Section 4.9).

In conclusion, the iron contained in unprocessed soya beans is poorly absorbed, but the bioavailability may be increased by specific processing methods, e.g., removing part of the phytate content [100, 116].

### 4.7. Milk Proteins

Bovine milk contains almost no iron [113, 115]. From a semisynthetic meal of low iron bioavailability (mean absorption 1.4%) containing ovalbumin as the protein source, substitution of the ovalbumin protein with animal meat protein resulted in a significant increase in iron absorption [62]. However, when the ovalbumin was substituted with protein from bovine milk and cheese, there was no increase in iron uptake [62], indicating that milk proteins have an inhibitory effect on iron absorption. In a subsequent study, the bovine milk proteins, casein and whey, were also shown to inhibit iron absorption [118].

Clusters of phosphoserine residues in cow milk caseins bind ferric iron with high affinity [119]. Caseins inhibit iron absorption in humans, but hydrolysis of whole caseins lessens this effect and can improve iron absorption [118]. Altogether, cow milk proteins have a negative effect on iron uptake, and in whole milk and milk products, this effect can be enhanced by the presence of calcium in the milk and cheese (see Section 4.9).

However, the inhibitory effect of milk proteins and milk calcium on iron uptake, which has been shown in single-test-meal studies, cannot simply be interpolated into real-life dietary studies. A moderate intake of milk (one glass of skimmed milk or yogurt once daily) consumed with a typical standard meal had no measurable effect on iron absorption when compared with the reference meal without milk [51, 52]. Likewise, nonfat milk consumed with a cereal-based diet had no influence on iron absorption in young women with small body iron reserves [120].

### 4.8. Egg Proteins

In a semisynthetic meal containing ovalbumin as the protein source, i.e., with low iron bioavailability, the substitution of ovalbumin with whole egg protein did not change mean nonheme iron uptake, which remained low at about 1.4% [62].

The absorption of nonheme iron from eggs in test meals was significantly lower when compared with the reference meal [121], and adding egg white to a test meal decreased nonheme iron uptake [122].

In *in vivo* rat studies, egg yolk decreased iron uptake [121] and other rat studies confirmed that egg yolk protein and egg yolk phosvitin decreased the absorption of the divalent minerals iron, calcium, and magnesium [123].

Almost all the iron in eggs is present in the yolk. The iron content in egg yolk varies from 2.7 to 5.9 mg/100 g [113, 115, 124]. Iron content in egg white is extremely low about 0.1 mg/100 g [113, 115, 124].

Another mechanism for the poor iron bioavailability in eggs could be the formation of insoluble iron sulfides. Both egg yolk and egg white contain sulfur incorporated into sulfur proteins. When eggs are boiled for a long time, the yolk's surface and subsequently its whole interior turns green and black due to the formation of iron sulfide, when iron from the yolk reacts with hydrogen sulfide from the egg white. Iron sulfide is not water soluble, and the iron in these compounds is not available for absorption [125].

Altogether, both egg yolk and egg white contain proteinaceous and other chemical compounds that decrease iron absorption [62, 121–123], and the iron in eggs has a poor bioavailability.

### 4.9. Calcium

The most calcium-rich nutrients are bovine milk and dairy foods produced from milk. Some green leafy vegetables such as spinach, broccoli, cabbage, okra, and some legumes like soya beans [113, 115, 126] also contain appreciable amounts of calcium.

Calcium inhibits iron absorption in single-test-meal settings [127], and the inhibition includes both nonheme and heme iron. The effect is partly dose related, being more dependent on the total amount of calcium administered than the calcium : iron molar ratio. In single-test-meal studies, iron uptake was inhibited by calcium supplements and by dairy products; the effect was depending on the simultaneous presence of calcium and iron in the intestine and also present when calcium and iron were given together in the fasting state [128–131].

However, the inhibitory effect of calcium on iron uptake demonstrated in single-test-meal studies is more complex to evaluate in daily-life dietary settings. A study in 6 European countries, comprising 1,604 adolescent girls and young women, demonstrated a significant negative association between dietary calcium intake, assessed by a three-day food record, and body iron status (serum ferritin) [132]. On the other hand, long-term intervention studies of calcium supplementation have failed to show any relevant reduction in body iron status (serum ferritin), even in women being at risk for low iron status; for review, see [127, 133].

Calcium inhibits the absorption of heme and nonheme iron to the same extent, which suggests that calcium interferes with the transport of iron through the enterocyte, probably at a stage in the absorption process, which is common for both heme and nonheme iron transport [130]. There may be an adaptive intestinal response to long-term calcium supplementation, generating a “normalization” of the iron absorption process. This hypothesis is supported by an *in vitro* study of Caco-2 cells, where the addition of calcium decreased the ferroportin expression at the basolateral membrane, resulting in a decreased iron efflux and increased cellular iron retention. However, after a few hours, DMT-1 and ferroportin expression increased again, suggesting a rebound effect [134].

### 4.10. Turmeric: Curcumin—Promoter or Inhibitor?

Curcumin is the active substance in turmeric, which is widely used as a spice, especially in the composite spice, yellow curry powder, and curry paste. Many naturopathic doctors/nutritionists/dieticians consider curcumin to be of benefit for patients with iron overload and haemochromatosis [135].

Curcumin contains polyphenolic compounds, which have antioxidative and anti-inflammatory properties [136]. Curcumin, a low-molecular-weight polyphenolic diketone, forms soluble iron complexes in aqueous solution [137], but the effect of curcumin on iron absorption is not clarified.

Due to its high content of polyphenols, it could be anticipated that curcumin would inhibit iron uptake, and some have even suggested that curcumin has iron-chelating properties that impair iron absorption [138]. However, in test meal studies, using stable iron isotopes, turmeric had no influence on iron absorption, despite a high content of polyphenols [111]. In contrast, chili induced a decrease in iron absorption, even though it had a lower content of polyphenols [111].

Regular ingestion of curcumin is recommended by many nutritionists being in favor of anti-inflammatory diets. However, from an iron absorption point of view, curcumin may in the long term have an indirect enhancing effect on iron uptake because curcumin inhibits the synthesis of hepcidin through inhibition of the signal transducer and activator of transcription 3 (STAT3) transduction pathway. This effect has been demonstrated both in mice [139] and in humans [140]. Although curcumin may be of benefit for patients with iron overload due to its antioxidant and anti-inflammatory properties, more studies are needed to clarify the final role of curcumin in human iron metabolism.

## 5. Discussion

In human iron metabolism, the focus has for many years been on iron deficiency, which still is frequent in certain parts of the populations all over the world, even in the Western countries [141], and a search on the Internet reveals a tremendous amount of information about the prevention and treatment of iron deficiency, which also includes a large number of nutritional and dietary advices, food recipes, and cookbooks.

In the last decades, the advances in genetics and molecular biology have brought much new information about the nature and prevalence of iron overload disorders, including the different forms of genetic haemochromatosis, which is emerging as a significant health concern among populations in Western countries [4–7, 9, 10]. Lifestyle changes have introduced new forms of iron overload, such as the metabolic syndrome with fatty liver and/or dysmetabolic iron overload syndrome [142] or other liver diseases with iron overload due to impaired hepcidin expression and low serum hepcidin levels [143].

So far, the interest for dietary intervention in haemochromatosis has been modest. Basically, patients with haemochromatosis are advised a healthy, balanced diet and to avoid iron-fortified food items and vitamin-mineral supplements containing iron. However, during the recent years, there appears to be an increasing interest for the effect of dietary intervention, from both treaters and patients. A number of cookbooks with dietary recipes for haemochromatosis patients [144, 145] have been published, but in the evaluation and treatment of these patients, at least in the Danish healthcare system, nutritional advices/recommendations for dietary modulations by a nutritionist/dietician are not routinely implemented.

According to Moretti et al. [3], restricting the absorption of dietary iron might reduce the annual amount of blood, which has to be removed by 0.5–1.5 L, depending on the penetrance of the disorder and the degree of dietary intervention.

This conclusion is supported by a study of extrinsic factors influencing the expressivity (serum ferritin and serum transferrin saturation levels) of the HFE-variant phenotypes in Danish men. The expressivity was enhanced by alcohol and meat consumption and decreased by milk and egg consumption as well as the official donation of blood in blood banks [146].

The Danish Haemochromatosis Association [147] has performed a small dietary survey among the members, which revealed that almost all patients are omnivorous, consuming mammalian and avian meat several times a week, and most of them taking alcoholic beverages with/without meals daily or several times a week [148]. Only few patients were offered dietary intervention. After consulting a dietician, a few have converted to a predominantly vegetarian diet, which has had a clear reducing impact on the rate of iron accumulation (assessed by the time-related increase in serum ferritin) according to the patients' own statements (Milman NT, personal communication). Some patients with a relatively high daily alcohol consumption who abstained from alcohol reported a marked decrease in the rate of iron accumulation (Milman NT, personal communication).

Almost all nutritional papers and cookbooks state that oxalic acid/oxalates are strong inhibitors of iron absorption although the documentation is controversial [91, 92]. The latest human study from 2008 found no effect of oxalate on iron absorption [93], so probably the effect of oxalic acid/oxalates on iron uptake has been exaggerated or misinterpreted and nutritional recommendations concerning oxalic acid/oxalate should be revised.

There is also controversy concerning the effect of turmeric/curcumin, which may be both an inhibitor and/or promoter of iron absorption [111, 135–140]. Some authors claim that it acts as an iron chelator—but at the same time, it reduces the expression of hepcidin; further studies are necessary to clarify which of these two effects are prevailing.

A diet designed for persons with iron overload due to excessive intestinal iron absorption should contain a minimum of nutrients and/or compounds with a high iron content, which at the same time contribute to increase iron absorption. The intake of mammalian and avian meat protein (beef, pork, and poultry) containing nonheme and heme iron plus meat factors should be restricted and/or partly substituted by fish meat, which has a lower content of both nonheme and heme iron. The intake of alcohol should be low. Ascorbic acid supplements and fruits and juices rich in ascorbic acid and other organic acids, which may increase iron absorption, should be consumed between the main meals [149]. Lactic acid fermented foods (except milk products) should be reduced or avoided [42, 49, 51].

The diet should be rich in nutrients and compounds that decrease iron absorption, especially the strong inhibitors phytates in cereals and polyphenols in plants and teas. Fluid intakes to the main meals should preferably be in the form of tea, milk, or water—not alcoholic drinks. Regular tea drinking at the main meals significantly decreases the rate of iron accumulation and the number of phlebotomies in the maintenance phase [110].

These dietary premises are best fulfilled by a predominantly plant-based, vegetarian diet, which also contains milk products, eggs, and fish meat—a “vegetarian-lacto-ovo-pescetarian” diet [149]. The dietary advices should be implemented as soon as the diagnosis of haemochromatosis has been confirmed. It is difficult to evaluate the dietary effect in the induction phase, and most dietary studies have therefore evaluated the effect in the maintenance phase [3]. However, in the induction phase, the frequent phlebotomies, rapidly diminishing iron overload, probably has a strong stimulating effect on iron absorption, which is an argument for early dietary intervention. In healthy persons, the maximal erythropoietic response to phlebotomy-induced anemia is a 200% increase over basal erythropoietic rate [150]. In haemochromatosis patients, the erythropoiesis works in a normal way and most patients regain their habitual haemoglobin level in a few weeks.

By each phlebotomy of 400–500 ml whole blood, approximately 130–140 g of protein is lost from the body. This is equivalent to two-day needs for protein [19]. The protein loss is highest in the induction phase of the treatment with weekly phlebotomies. It is therefore important to consume adequate protein—preferably from milk, whey, eggs, and protein-rich plants, e.g., soya beans, as the proteins from these nutrients by themselves inhibit iron uptake. The consumption of animal protein should be restricted and preferably be from fish and young poultry with a relatively low content of heme iron [149].

In healthy subjects, the composition of the diet and body iron status are the two main factors determining iron bioavailability and absorption. The bioavailability factor is usually estimated in relation to “reference subjects” with low or no body iron reserves [151]. In haemochromatosis, due to the dysregulation of iron absorption, which is permanently upregulated, being by and large independent of body iron stores, the patients are therefore in a state mimicking a continuous marked “iron deficiency” or “iron craving,” which will tend to obtain maximum iron availability from almost any diet.

The prediction of iron bioavailability using algorithms in healthy individuals can only predict high, medium, and low bioavailability [151, 152]. The bioavailability of iron from Western-type diets is, on average, 15% with a range of 14–17%. Iron in diets with little meat (50–100 g/day), occasionally with fruit or vegetables consumed with the main meals, and more whole-grain cereals, is 10–12% bioavailable. Vegetarian diets have the lowest bioavailability ranging from 5 to 12% [17]. Although dietary habits in Western countries have changed somewhat since the 1990s, the overall dietary iron intake in women and men in Europe has been quite constant from the 1990s until now [24].

Another aspect of dietary modulation could be to supplement the main meals with some of the known natural inhibitors of iron absorption in balanced amounts, e.g., specific phytates, specific polyphenols, or specific calcium compounds or alternatively artificial nonabsorbable food iron chelators. These dietary approaches have not been investigated.

Finally, it must be emphasized that dietary intervention should be implemented on a voluntary basis, after targeted dietary counselling and education of the patient.

## 6. Conclusions

In patients with genetic haemochromatosis, intestinal iron absorption is enhanced due to a decreased expression of hepcidin. Overall, we have detailed knowledge of the mechanisms of iron absorption to design dietary interventions that may decrease iron intake and iron uptake. Dietary modifications that lower iron intake and iron bioavailability may provide additional measures to limit iron uptake from the diet. This could stimulate the patients' active cooperation in the treatment of their disorder. However, there is a need to perform controlled studies of the effect of targeted long-term dietary intervention and to evaluate the effect of dietary supplements containing specific inhibitors of iron absorption.

## Figures and Tables

**Table 1:** Promoters and inhibitors of iron absorption.

Promoters	References no.	Inhibitors	References no.
Gastric acid	[33–35]	Gastric acid inhibitors	[80–84]
		Antacids	[34, 85–87]
*Organic acids*		Oxalic acid-Oxalates?	[88–93]
Ascorbic acid	[37–48]
Citric acid?	[38, 39, 42]
Malic acid?	[39, 42]
Tartaric acid	[42]
Lactic acid	[42, 49–60]
		Phytic acid-phytates	[53–60, 94–101]
Animal protein: meat factors	[16, 61–63]	Polyphenols	[102–111]
Heme: heme iron	[30, 64–68]	Soya bean proteins	[31, 32, 38, 99, 112–117]
Alcohol: ethanol	[69–79]	Milk proteins	[51, 52, 62, 113, 115, 118, 120]
		Egg proteins	[62, 113, 115, 121–125]
		Calcium	[113, 115, 126–134]
Turmeric: curcumin?	[111, 135–137, 139, 140]	Turmeric: curcumin?	[111, 135–138]

## Data Availability

This review is based on literature searches in the PubMed and Google Scholar databases as well as literature references cited in published articles, review papers, and books on iron metabolism. The data used to support the findings of this study are included within the article.
